# S-Nitrosylation of Paraxonase 1 (PON1) Elevates Its Hydrolytic and Antioxidant Activities

**DOI:** 10.3390/biom12030414

**Published:** 2022-03-07

**Authors:** Hanin Hajouj, Ali Khattib, Dana Atrahimovich, Sanaa Musa, Soliman Khatib

**Affiliations:** 1 Department of Natural Compounds and Analytical Chemistry, MIGAL—Galilee Research Institute, Kiryat Shmona 11016, Israel; hanin.hajouj@gmail.com (H.H.); alikhattib94@gmail.com (A.K.); dana.atr@gmail.com (D.A.); sanaa@migal.org.il (S.M.); 2 Department of Biotechnology, Faculty of Science, Tel-Hai College, Upper Galilee 12210, Israel

**Keywords:** S-nitrosylation, rePON1, HSA-NO, atherosclerosis, cardiovascular disease

## Abstract

Covalent binding between nitric oxide (NO) and a protein’s free thiol group (SH) is termed protein S-nitrosylation. Protein S-nitrosylation is involved in cellular regulation mechanisms that underlie a wide range of critical functions, such as apoptosis, alteration of enzyme activities, and transcription-factor stability. Impaired protein S-nitrosylation is associated with a growing list of pathophysiological conditions, such as cardiovascular disease, multiple sclerosis, pulmonary hypertension, and sickle cell disease. The enzyme paraoxonase 1 (PON1) binds to high-density lipoprotein to provide many of its antiatherogenic properties. The enzyme has a strong antioxidant capacity, which protects fats, lipids, and lipoproteins from oxidation, in addition to breaking down oxidized fats. We investigated the effect of S-S transnitrosylation on PON1 activities. Incubation of recombinant PON1 (rePON1) with nitrosylated human serum albumin (HSA-NO) resulted in S-nitrosylation of about 70% of the rePON1, as measured by Q-TOF LC/MS. S-nitrosylation significantly increased rePON1 hydrolytic activities. It also increased rePON1’s ability to inhibit low-density lipoprotein oxidation induced by Cu^2+^. Finally, it increased the enzyme’s penetration into macrophage cells by 31%. Our findings suggest that S-nitrosylation of rePON1 improves its biological functions which may positively affect atherosclerosis disease progression.

## 1. Introduction

Proteins’ physical and biological properties can change as a result of their interaction with elements in their environment. Covalent post-translational modifications are of great importance in increasing proteomic diversity [1,2]. S-nitrosylation of proteins—the covalent binding of nitric oxide(NO) molecule to the free thiol group (SH) of a protein to form S-nitrosothiols (SNOs)—is one such post-translational modification that plays a key role in human health and disease states by affecting proteins’ structure, stability, and roles [1,3]. S-nitrosylation of proteins is important in the regulation and control of their activities, including ion channel, receptor, respiratory-related and enzymatic activities, and in improving their circulation in the blood [4,5]. Several proteins related to the cardiovascular system have been reported to undergo S-nitrosylation and affect many important processes in the body, as well as health and disease states. Defects in NO formation and transfer to proteins are associated with cardiovascular disease and arterial hypertension [6,7].

In addition to direct binding of the NO molecule to the thiol group, protein S-nitrosylation can occur through the transfer of a NO group from a protein or molecule that contributes NO (NO donor) to another protein that receives NO (NO acceptor), a process called S-S protein transnitrosylation [8]. Human serum albumin (HSA) is known for its ability to bind free NO released from endothelial cells and transfer it to other proteins in the circulation [2].

Atherosclerosis is a chronic disease of the blood vessels, characterized by the accumulation of fats and oxidized fats in the intima of the arterial wall, along with cells originating in the bloodstream and from the artery itself, which together form plaques that inhibit blood flow and clots that block the artery and cause a heart attack or stroke [9].

High-density lipoprotein (HDL) particles play a major role in preventing atherosclerosis development because of their anti-atherosclerotic properties—collecting excess cholesterol and returning it to the liver, exhibiting antioxidant and anti-inflammatory properties, and improving endothelial cell function [6,10,11]. Paraoxonase 1 (PON1) is a glycoprotein enzyme that is synthesized in the liver and transferred to the bloodstream, where it binds to the HDL particles and confers many of their anti-atherosclerotic properties [12,13]. The enzyme has a strong antioxidant ability, which, in addition to breaking down oxidized fats, protects fats, lipids, and lipoproteins from oxidation [14,15]. PON1 is known for its various hydrolytic activities, including lactonase, esterase, and paraoxonase activities [16]. Although the enzyme’s natural substrate is not known, studies suggest that lactonase is its main natural activity [16,17]. PON1 is also known for its ability to degrade oxidized fats, prevent HDL and low-density lipoprotein (LDL) oxidation, and mediate macrophage flow [18,19]. There is epidemiological evidence of low PON1 activity being associated with an increased risk of cardiovascular events and heart disease [2]. In vitro and model animal studies have provided strong support for the conclusion that PON1 protects against the development of atherosclerosis by protecting HDL and LDL from oxidation and reducing oxidative stress [20,21,22]. PON1 contains a free thiol group on the amino acid cysteine 284 (Cys-284) which can undergo various modifications, including nitrosylation [14,16].

In a previous study, we showed that Cys-284 of recombinant PON1 (rePON1) undergoes nitrosylation rePON1-NO after exposing the protein to two different NO donors, S-nitrosoglutathione (GSNO) and nitrosylated HSA (HSA-NO) [2]. Here we examine the effect of rePON1 S-nitrosylation on its hydrolytic activities, its ability to inhibit LDL oxidation, and its penetration of macrophage cells toward determining the effect of rePON1 S-nitrosylation on atherosclerosis.

## 2. Materials and Methods

### 2.1. Materials

GSNO, glutathione, HSA, cholesterol acyltransferase inhibitor (Sandoz 58-035), buffers, fluorescein isothiocyanate (FITC), neocuproine, dithiothreitol (DTT), solvents, and reagents were purchased from Sigma-Aldrich, Jerusalim, Israel. BODIPY-cholesterol was purchased from Avanti Polar Lipids (Alabaster, AL, USA). Dulbecco’s Modified Eagle Medium (DMEM) containing 10% fetal bovine serum (FBS) and 1% penicillin–streptomycin was from Biological Industries (Beit HaEmek, Israel). J774A.1 cells were purchased from the American Tissue Culture Collection (ATCC, Rockville, MD, USA). HEN buffer (50 mm HEPES buffer, 1 mm EDTA, 0.1 mm neocuproine, pH 7), Tris pH 8, phosphate-buffered saline (PBS) pH 7.4, and binding buffer consisting of Tris-buffered saline (50 mm Tris-HCl, 150 mm NaCl) pH 7.5, were prepared in the laboratory.

### 2.2. Recombinant PON1 (rePON1)

rePON1 was purchased from the Weizmann Institute of Science (Rehovot, Israel). rePON1 was generated in *Escherichia coli* by directed evolution as described previously [23]. rePON1 storage buffer (50 mm Tris, pH 8.0, 50 mm NaCl, 1 mm CaCl_2_, and 0.1% (*v*/*v*) tergitol) was supplemented with 0.02% (*w*/*v*) sodium azide, and storage was at 4 °C.

#### 2.2.1. S-Nitrosylation of rePON1 by HSA-NO

S-nitrosylation of rePON1 was generated as described previously [2]. HSA (0.1 mm) was dissolved in PBS buffer, DTT (final concentration 1 mm) was added, and the solution was incubated at 37 °C for 10 min. Excess DTT was removed by filtration through an Amicon ultracentrifuge filter device (MW cutoff 10,000 Da, 37 °C, 4000 rpm (3220× *g*). The protein was then redissolved in HEN buffer. GSNO (5 mm) was added to the solution and incubated at 37 °C for 1 h in the dark. Excess GSNO was removed by Amicon filtration and centrifugation, and the solution was injected into the Quadrupole Time-Of-Flight (Q-TOF) LC/MS to measure percent nitrosylation of HSA. RePON1 (1.25 µm in HEN buffer) was incubated with the HSA/HSA-NO mixture (10 µm, 20 µm, 40 µm) for 1 and 3 h at 37 °C and percent S-nitrosylation of rePON1 was determined by Q-TOF LC/MS. The experiment was performed three times, with three technical replicates each time.

#### 2.2.2. LC/MS Analysis

Reversed-phase LC separation of rePON1/rePON1-NO was carried out in a 1290 Infinity LC system (Agilent Technologies, Santa Clara, CA, USA). The protein was separated in a C-18 reversed-phase column (Zorbax 300 SB-C182, 1 × 50 mm 1.8 µm) with a 10-min gradient of solvent A (DDW with 0.1% formic acid) and solvent B (acetonitrile and 0.1% formic acid) started at 0 min with 5% solvent B and raised to 90% solvent B at 2 min and 98% solvent B at 8 min at a flow rate of 0.2 mL/min. The LC eluent was introduced directly into the electrospray ionization (ESI) source connected to the Q-TOF MS.

### 2.3. MS

Protein analyses were carried out on a UHD accurate-mass Q-TOF LC/MS 6540 (Agilent Technologies, Santa Clara, CA, USA). The ESI capillary voltage was set to 3500 V, fragmentor 150 V, gas temperature 300 °C, gas flow 9 mL/min, and nebulizer 40 psig. The mass spectra (*m*/*z* 100–3000) were acquired in positive-ion mode. Deconvolution of the *m*/*z* spectra to the exact mass of the protein was generated using the maximum entropy algorithm with a mass range of 35,000.0–45,000.0 Da, mass step 1.0 Da, S/N threshold 50, and *m*/*z* range 800–2500.

#### 2.3.1. RePON1-NO Lactonase and Aryl Esterase Activity

Lactonase and aryl esterase activities of rePON1 with and without SNO were measured using dihydrocoumarin and phenylacetate assays, respectively. The enzyme was diluted 10-fold to a final concentration of 5 µ/mL in HEN buffer pH 7, then 10 µL was transferred to a UV microplate containing Tris-HCl buffer solution pH 8 (90 µL). The volume in the wells was brought to 200 µL by addition of 100 µL dihydrocoumarin or phenylacetate substrate solution at a final concentration of 1 mM to test lactonase and aryl esterase activity, respectively. The catalytic activity of rePON1 was measured in a spectrophotometer (Tecan Infinite M200 PRO, Mannedorf, Switzerland) every 0.5 min for 10 min at 270 nm caused by the formation of phenol product. A molar extinction coefficient of 1300 M^−1^ cm^−1^ was used for the calculation of enzyme activity [24]. Self-decomposition of the substrates was also measured and subtracted from the samples. The result was multiplicate by 20 because of the dilution. The experiment was performed three times, with three technical replicates each time. The results are presented in activity units, defined as the ability of the enzyme to break down dihydrocoumarin or phenylacetate (1 µmol) per min. under the given conditions

#### 2.3.2. RePON1-NO Paraoxonase Activity

A 10-µL aliquot of the enzyme sample was placed in a UV microplate containing 90 µL glycine reaction buffer (50 mm glycine/NaOH pH 10.5 + 1 mm CaCl_2_) and brought to 200 µL by adding 100 µL paraoxon substrate solution at a final concentration of 2 mm. The catalytic activity of rePON1 was measured in a spectrophotometer (TECAN Infinite M200 PRO, Mannedorf, Switzerland) every 0.5 min for 10 min at 412 nm caused by 4-nitrophenol formation. A molar extinction coefficient of 18,000 M^−1^ cm^−1^ was used for the calculation of enzyme activity [24]. Self-decomposition of the substrate was also measured and subtracted from the samples. The result was multiplicated by 20 because of the dilution. The experiment was performed three times, with three technical replicates each time. The results were presented in activity units, defined as the ability of the enzyme to break down paraoxon (1 µmol) per min under the given conditions.

#### 2.3.3. Extraction of LDL from Human Serum by Ultracentrifugation

LDL was isolated from human plasma by ultracentrifugation (37,000 rpm (3220× *g*) for 48 h, at 4 °C with amplification speed 5 and stopping speed 7). Prior to the experiments, LDL/HDL was dialyzed twice in PBS buffer pH 7.4, 1 h each time, and once more overnight in PBS at 4 °C.

#### 2.3.4. RePON1-NO Protection of LDL from Oxidation

RePON1 (50 µg/mL) in HEN buffer was incubated with GSNO (100 µm) for 5 h. GSNO was removed from the solution by Amicon filtration and centrifugation (10,000 Da, 37 °C, 4000 rpm (3220× *g*) with PBS buffer, and the protein concentration was measured. Then 5 or 10 µL rePON1 was added to the microplate at a concentration of 100 µg/mL, with 10 µL LDL (0.05 mg protein/mL) and 10 µL CuSO_4_ (100 µm) and brought to 100 µL with PBS buffer. LDL oxidation was tested by monitoring the formation of conjugated dienes at 234 nm every 10 min for 2.5 h at 37 °C in a SpectraMax M2 reader (Molecular Devices, San Jose, CA, USA). The experiment was performed three times.

#### 2.3.5. Treatment of J774A.1 Macrophage Cells

J774A.1 macrophage cell lines were incubated in an incubator at 37 °C and 5% CO_2_ until full cell density for the experiment. The cells were collected and centrifuged at 175× *g* for 7 min. The resulting output was suspended in 5 mL DMEM containing 10% FBS and 1% penicillin–streptomycin solution, and the cells were counted in a hemocytometer, with 40 µL of Trypan blue added to 40 µL of the cell sample to eliminate dead cells in the count; cell number (X) was calculated according to the formula: X $\times 10^{4}\times \frac{60}{40}\;$=$\;\frac{Number\;of\;cells}{\mathrm{mL}}$ (X = average of three biological replicates, $\frac{60}{40}$ = dilution of sample volume with Trypan blue).

#### 2.3.6. RePON1-NO Influx into Macrophages

RePON1 (2 mg/mL) was incubated with FITC (0.25 mg/mL) for 2 h. Free FITC was removed by dialysis 1:1000 in PBS buffer (cutoff 10,000 Da, 4 °C, the buffer was replaced after 1, 3 h and left for 24 h), and the protein concentration was measured. The enzyme was S-nitrosylated by incubation with GSNO at 50-fold the concentration of the protein for 3 h, then GSNO was removed by Amicon filtration (cutoff 10,000 Da, 37 °C, 4000 rpm (3220× *g*)) in PBS buffer. RePON1 and S-nitrosylated rePON1 (concentration of 50 µg/mL) were added to J774A.1 mouse macrophage cells (250,000 cells/well) treated with DMEM containing 10% FBS and 1% penicillin–streptomycin solution. Then the cells were incubated at 37 °C for 16 h in the dark and analyzed by fluorescence-activated cell sorting using the NovoExpress program. The experiment was performed three times.

#### 2.3.7. Statistical Analysis

Statistical analysis was carried out using GraphPad prism 8.0.1 and NovoExpress software, and the Excel 2016 program. One-way ANOVA was used to compare the means of groups. Each experiment was repeated at least three times separately. Results are presented as means ± SEM.

## 3. Results

### 3.1. RePON1 S-Nitrosylation Using HSA-NO as NO Donor

**Figure 1:**
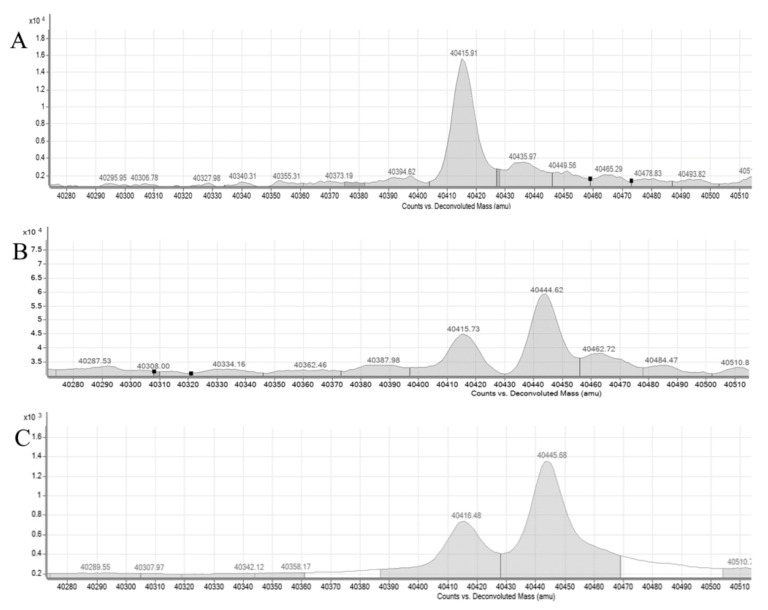
Mass spectra of rePON1 and rePON1-NO obtained by Q-TOF LC-MS. (**A**) Mass spectrum of rePON1 (1.25 μm) incubated in HEN buffer (50 mm HEPES buffer, 1 mM EDTA, 0.1 mm neocuproine, pH 7). (**B**,**C**) Mass spectrum of rePON1 (1.25 μm) after incubation with HSA-NO (10 μm) in HEN buffer for 1 h (**B**) and 3 h (**C**). Data are mean ± SEM of triplicate wells and are representative of three independent experiments. Statistical analysis by one-way ANOVA and GraphPad prism 8.0.1 software.

The ability of rePON1 to undergo S-nitrosylation on Cys-284 was examined. RePON1 was incubated with different amounts of HSA or HSA-NO and analyzed by Q-TOF LC/MS. Deconvolution of the *m*/*z* spectra of rePON1 showed a single peak with a mass of 40,415 (Figure 1A). Upon incubation with HSA-NO for 3 h, the deconvoluted mass spectra presented two peaks with masses 40,415 and 40,444 *m*/*z* for rePON1 and rePON1-NO, respectively. The 29-unit difference corresponded to the addition of NO to rePON1. The ratio of rePON1-NO to total rePON1 was measured from the area obtained under each deconvoluted peak. We observed a dose-dependent and time-dependent increase in the percentage of rePON1-NO, 53% after 1 h incubation with 10 μM HSA-NO and peaking at 66% after 3 h incubation with 40 μm HSA-NO (Figure 1 and Table 1). This experiment verified that HSA-NO could transfer its NO group to rePON1 via S-S transnitrosylation.

### 3.2. Hydrolytic Activities of the S-Nitrosylated rePON1

**Figure 2:**
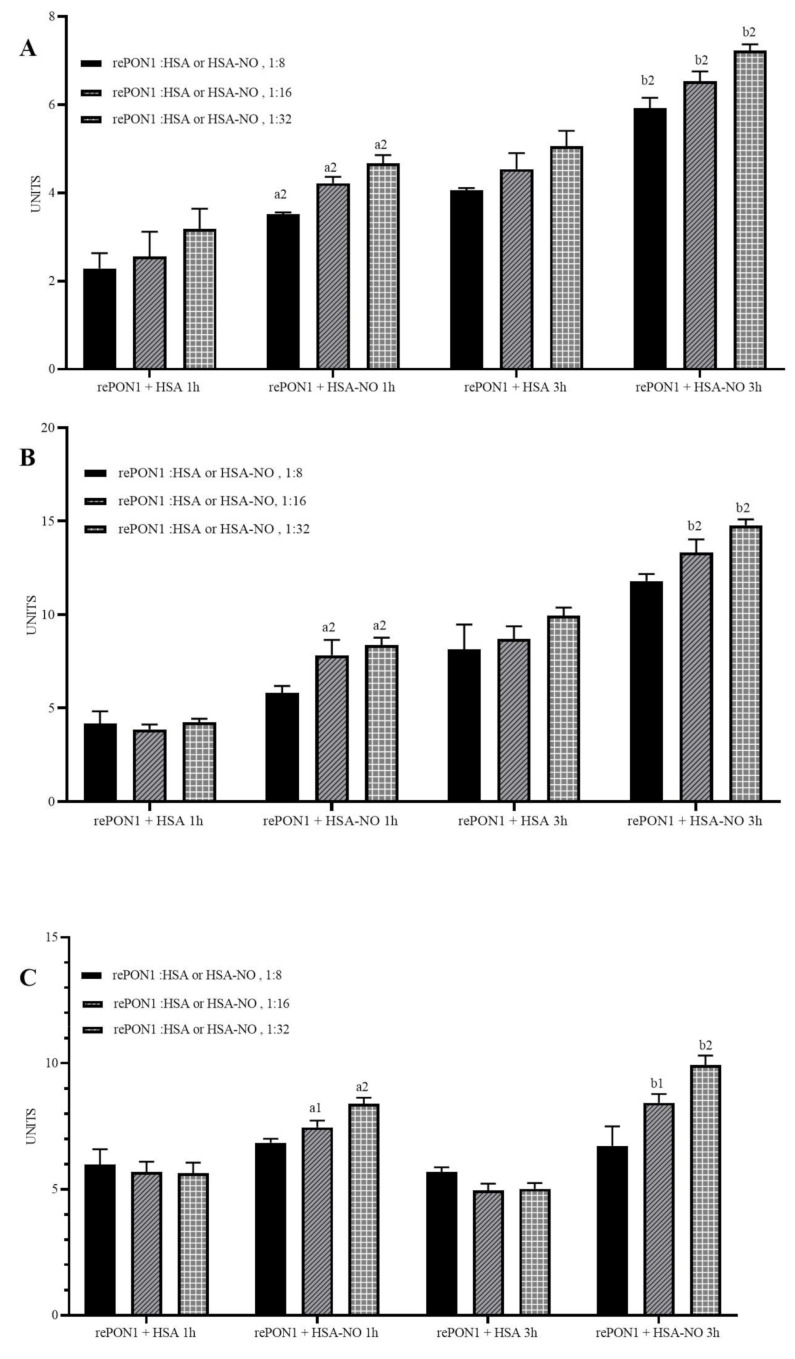
S-nitrosylation of rePON1 with HSA-NO as the donor increases the enzyme’s hydrolytic activities. RePON1 (1.25 µm) was incubated with different concentrations of HSA or HSA-NO (10, 20, 40 µm) for 1 and 3 h at 37 °C in HEN buffer (50 mm HEPES buffer, 1 mm EDTA, 0.1 mm neocuproine, pH 7). (**A**) Lactonase activity (5 µg/mL rePON1). (**B**) Esterase activity (5 µg/mL rePON1). (**C**) Paraoxonase activity (50 µg/mL rePON1). Each experiment was repeated separately three times with three replicates each time; a1 or b1 above a bar indicates *p* < 0.05 relative to rePON1 incubated with HSA for 1 and 3 h, respectively; a2 and b2 = *p* < 0.0001. Statistical analysis by one-way ANOVA and GraphPad prism 8.0.1.

Incubation of rePON1 with HSA-NO for 1 h significantly increased its lactonase activity by approximately 50%, 70%, and 50% for rePON1:HSA-NO ratios of 1:8, 1:16, 1:32, respectively, compared to rePON1 incubated with HSA at the same ratios; incubation for 3 h increased its lactonase activity by approximately 40–50%, relative to incubation with HSA (Figure 2A).

Incubation of rePON1 with HSA-NO for 1 h significantly increased its aryl esterase activity by approximately 100% for rePON1:HSA-NO ratios of 1:16 and 1:32 compared to rePON1 incubated with HSA at the same ratios; incubation for 3 h at these ratios significantly increased its aryl esterase activity by approximately 50% relative to incubation in HSA (Figure 2B).

Incubation of rePON1 with HSA-NO for 1 h significantly increased its paraoxonase activity by approximately 30% and 50% for rePON1:HSA-NO ratios of 1:16 and 1:32, respectively, compared to rePON1 incubated with HSA at the same ratios; incubation for 3 h significantly increased its paraoxonase activity by approximately 70% and 100%, respectively, relative to incubation in HSA (Figure 2C).

### 3.3. Effect of S-Nitrosylation on rePON1 Antioxidant Activity: Protection of LDL from Oxidation

**Figure 3:**
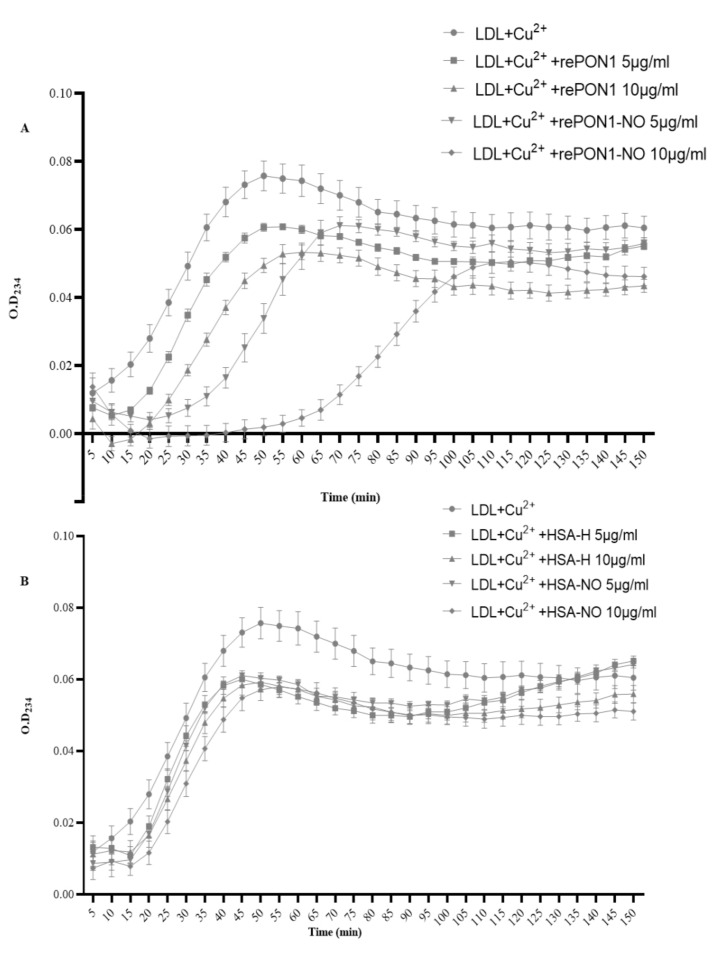
Effect of rePON1-NO and HSA-NO on LDL oxidation induced by Cu^2+^. (**A**) RePON1 (5 and 10 μg/mL) and rePON1-NO (5 and 10 μg/mL) and (**B**) HSA (5 and 10 μg/mL) and HSA-NO (5 and 10 μg/mL) were incubated with LDL (0.05 mg protein/mL) and Cu^2+^ (10 μm). Absorbance at 234 nm was measured every 5 min for 2.5 h. Data are mean ± SEM of three representative independent experiments. Statistical analysis by one-way ANOVA and GraphPad prism 8.0.1 software.

The ability of rePON1-NO to protect LDL from oxidation induced by Cu^2+^ was examined. RePON1 and rePON1-NO (55% S-nitrosylated as detected by Q-TOF LC/MS) were incubated with LDL and Cu^2+^_._ Oxidation of LDL fatty acids produced conjugated dienes, and their absorbance at 234 nm was monitored every 5 min for 2.5 h. RePON1 delayed LDL oxidation rate in a dose- and time-dependent manner (Figure 3A). RePON1-NO showed better prevention of LDL oxidation than rePON1 (Figure 3A).

The lag time of LDL oxidation increased by 10 and 15 min after incubation with 5 μg/mL and 10 μg/mL rePON1, respectively. After incubation with 5 μg/mL and 10 μg/mL rePON1-NO, lag time increased by 30 and 60 min, respectively.

RePON1 increased the propagation phase of LDL oxidation by 10% at a concentration of 5 μg/mL and decreased it by 18% at a concentration of 10 μg/mL. RePON1-NO decreased the propagation phase of LDL oxidation by 24% and 62% at 5 μg/mL and 10 μg/mL, respectively.

To ensure that LDL oxidation was actually attenuated by incubation with rePON1 and rePON1-NO, we incubated LDL with HSA and HSA-NO: neither affected LDL oxidation (Figure 3B).

### 3.4. Effect of S-Nitrosylation of rePON1 on Its Influx into Macrophage Cells

**Figure 4:**
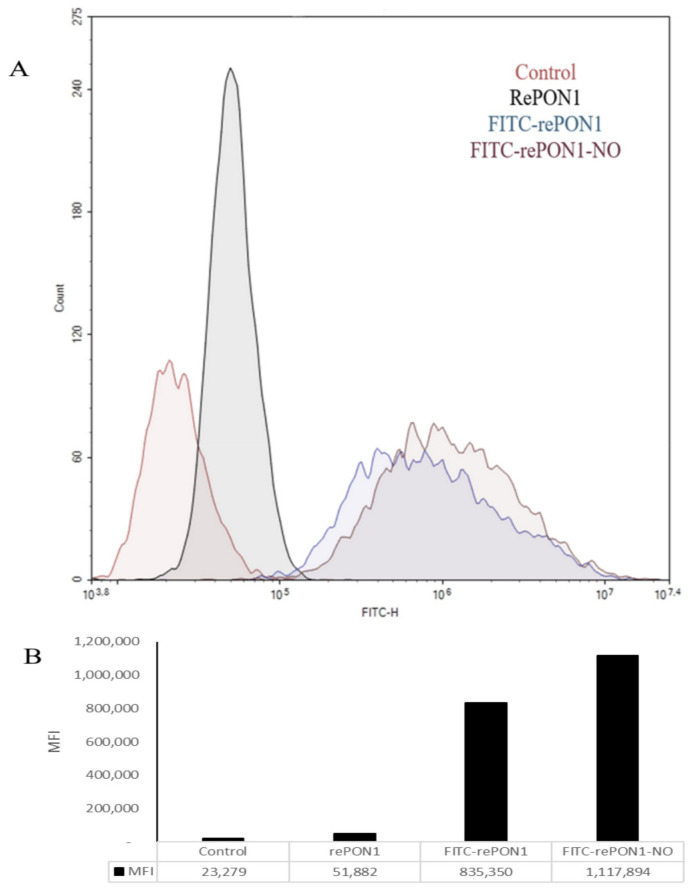
FITC-rePON1-NO influx into macrophage cells compared to FITC-rePON1. RePON1 was labeled with FITC and incubated with GSNO for 3 h to obtain 50% rePON1 S-nitrosylation. Then, rePON1-NO was added to J774A.1 macrophages, incubated at 37 °C for 16 h and analyzed by FACS. (**A**) Histogram shows the shift in mean fluorescence intensity (MFI) in a representative experiment. Control (cells without treatment) in red, rePON1 without FITC as a negative control in black, rePON1 with FITC in blue, and rePON1-NO with FITC in purple. (**B**) MFI of a representative experiment. Results presented using NovoExpress and GraphPad by prism 8.0.1 software.

Our laboratory studies have recently shown that rePON1 enters macrophages through endocytosis [22,25,26]. RePON1 interacts specifically with macrophages and is internalized into the cell cytoplasm, where it may protect the macrophages from oxidation and prevent foam cell formation. We examined the effect of rePON1 S-nitrosylation on its entry into macrophages (rePON1 influx). RePON1 was labeled with fluorescein isothiocyanate (FITC) and nitrosylated by GSNO to obtain 50% rePON1-NO. Then it was added to J774A.1 macrophage cells, and its ability to enter the macrophages compared to rePON1 was analyzed by FACS using the NovoExpress program. Figure 4 shows a 31% higher mean fluorescence intensity (MFI) for FITC-PON1-NO vs. FITC-PON1 in the macrophages, indicating better penetration of the former.

## 4. Discussion

Post-translational modifications play a major role in human health and disease states by affecting protein structure, stability, and function [1,3]. S-nitrosylation of proteins is an important covalent modification in the regulation and control of protein activity and acts as a basic mechanism in intracellular signaling (signal transmission), DNA repair, regulation of ion channels, and apoptosis [27,28]. Defects in NO formation or in its transfer to proteins are associated with cardiovascular disease and arterial hypertension [6,7].

Glutathione and HSA are known for their ability to bind free NO released from endothelial cells and transfer it to other proteins through S-S transnitrosylation [5]. The GSNO molecule has many positive activities and is very effective and efficient in maintaining the NO molecule and transferring it to other proteins when needed [29].

HSA is a plasma protein that acts as a carrier and reservoir of NO molecules produced in endothelial cells, transferring them to proteins with a free thiol, thereby freeing its own thiol. HSA is the most common plasma protein with a long lifespan as a NO donor compared to other donors [30]. Several in vitro and in vivo studies have revealed HSA-NOs’ potential to interact with exogenous drugs, thereby affecting their pharmacokinetic properties and half-life in the blood, reducing their toxicity, and improving their achievement of a specific target aim [31]. Few studies have examined the effect of NO-group donors, such as HSA-NO, on proteins associated with atherosclerotic development. Here, we examined HSA-NO’s ability to transfer NO to the antiatherogenic enzyme rePON1 and the effect of this nitrosylation on the enzyme’s atherosclerosis-related properties.

Protein–protein transfer of NO was observed. The literature shows that positively or negatively charged side residues of amino acids present in the thiol environment, environmental hydrophobicity, pKa of the thiol group, and the oxidative state in which it is present in the cell are important factors affecting the specificity of the NO-group transfer from one protein to another. Furthermore, important is the structural adjustment and the interaction between the target protein and the NO donor for NO transfer via the S-S transnitrosylation mechanism [30,31,32]. Thus, in a previous study in our laboratory, we examined HSA-NO and GSNO as NO donors for S-S transnitrosylation of rePON1. We found HSA-NO to be a 30% better NO donor to rePON1 than GSNO, and the S-nitrosylated rePON1 was stable for 1 week at 4 °C and for three days at 37 °C [2]. We quantified the percentage of protein nitrosylation by Q-TOF LC/MS, which has many advantages over other methods. It enables specific localization of the site of NO binding in a protein, and it is a rapid, straightforward, accurate, and easy-to-use method, with very high sensitivity [33]; moreover, it requires only a low concentration or small amount of protein compared to other methods [34].

Accordingly, we used Q-TOF LC/MS to quantify the percentage of rePON1 nitrosylation after incubation with HSA-NO at different concentrations and at various times. The percentage of rePON1 nitrosylation increased significantly with the HSA-NO dose and time of incubation. We then examined the biological effects of the modification.

Epidemiological evidence shows that low hydrolytic activity of PON1 is associated with an increased risk of cardiovascular disease [2]. We examined the effect of nitrosylation on the different hydrolytic activities of rePON1—lactonase, esterase, and paraoxonase—after incubation with HSA or HSA-NO for 1 and 3 h. Incubation of rePON1 with HSA-NO increased each of the examined activities relative to incubation with HSA alone (Figure 2). Research in our laboratory has shown that Cys-284 oxidation to sulfenic, sulfinic, and sulfonic acids inhibits PON1 activity [14], while a specific flavonoid—glabridin—that binds to the allosteric site of rePON1 near Cys-284 protects the enzyme from oxidation and preserves its hydrolytic activity [35]. Here we show that in contrast to oxidation, Cys-284 nitrosylation improves PON1’s hydrolytic activities. Thus, similar to protein phosphorylation, Cys-284 nitrosylation of PON1 may be expected to alter the protein conformation and change the molecular behavior of the enzyme. Similarly, it has been reported that protein S-nitrosylation enhances the activity of glycolytic enzymes in mice and enhances lactate dehydrogenase activity in Trichomonas vaginalis under iron deficiency [36].

The next step was to study the therapeutic effect of PON1 nitrosylation with respect to oxidation and atherosclerosis.

Oxidation of LDL accelerates the process of atherosclerosis formation while protecting LDL from oxidation reduces the risk of atherosclerosis. In vitro studies have shown that PON1 and PON1-bound HDL protect LDL from oxidation [20]. The effect of nitrosylation of rePON1 on its ability to protect LDL from oxidation was examined. Both rePON1 and rePON1-NO induced a concentration-dependent inhibition of LDL oxidation. RePON1-NO lengthened the lag time to oxidation and inhibited oxidation rate more efficiently than non-nitrosylated rePON1. This result reinforces the hypothesis that the modification of rePON1 via nitrosylation positively affects its antioxidant activities, including its ability to inhibit LDL oxidation, which may lead to a reduction in the risk of atherosclerosis and heart disease. Literature shows that S-nitrosylation is an emerging paradigm of redox signaling that could simultaneously control many proteins involved in divergent pathways. In addition, S-nitrosylation helps halt the production of reactive oxygen species, protects cellular proteins against oxidative damage, and propagates redox signaling. Moreover, a homeostatic level of S-nitrosylation could serve as an effective therapeutic intervention for certain diseases, warranting active investigation [37].

Although PON1 is a serum protein, it has been shown to accumulate in arterial walls during the development of atherosclerosis as part of a protective mechanism to prevent oxidation and disease progression [22]. These activities are of great importance in explaining the antioxidant and anti-inflammatory potential of this enzyme [37,38,39]. In addition, we previously showed that rePON1 enters macrophage cells through endocytosis: it interacts specifically with macrophages and is internalized into their cytoplasm, which may protect the macrophages from oxidation and prevent foam cell formation. Thus, the effect of nitrosylation on the entry of rePON1 into macrophages was examined. Nitrosylation of rePON1 enhanced its penetration of macrophages by 30% (Figure 4).

In summary, we present the effects of rePON1 S-S transnitrosylation by HSA-NO as the NO donor on the enzyme’s hydrolytic and antioxidant activities and on its penetration into macrophages. Our aim was to determine whether the modification by S-nitrosylation contributes to the antioxidative and antiatherosclerotic properties of the enzyme and its bound HDL particle. S-Nitrosylation of rePON1 improved its activity and its ability to protect LDL from oxidation. It also enhanced the enzyme’s penetration of macrophages, allowing this antioxidant protein to act from inside the cell, in addition to its antioxidant activity in the circulation. These findings confirm the positive effects of rePON1 S-nitrosylation, which may lead to a reduction in the oxidative level in circulation and decreased risk of atherosclerosis and cardiovascular disease.

## Figures and Tables

**Table 1:** S-nitrosylation after 1 or 3 h incubation with three different concentrations of HSA-NO donor.

Incubation Time	RePON1:HSA-NO Ratio of 1:8 (%)	RePON1:HSA-NO Ratio of 1:16 (%)	RePON1:HSA-NO Ratio of 1:32 (%)
1 h	53.35 ± 3.58	59.07 ± 0.37	57.6 ± 0.87
3 h	60.04 ± 3.25	64.13 ± 3.25	66.42 ± 2.13

## Data Availability

Data sharing is not applicable to the paper, all supporting data are included within the main article.
